# Capacity Increase Investigation of Cu_2_Se Electrode by Using Electrochemical Impedance Spectroscopy

**DOI:** 10.3389/fchem.2018.00221

**Published:** 2018-06-12

**Authors:** Xiuwan Li, Zhixin Zhang, Chaoqun Liu, Zhiyang Lin

**Affiliations:** Fujian Provincial Key Laboratory of Light Propagation and Transformation, College of Information Science and Engineering, Huaqiao University, Xiamen, China

**Keywords:** Cu_2_Se, capacity increase, electrochemical impedance spectroscopy, polymeric film, lithium ion battery

## Abstract

Cu_2_Se nanoflake arrays supported by Cu foams are synthesized by a facile hydrothermal method in this study. The Cu_2_Se materials are directly used as an anode for lithium ion batteries, which show superior cycle performance with significant capacity increase. Combining with previous reports and scanning electron microscope images after cycling, the capacity increase caused by the reversible growth of a polymeric film is discussed. Electrochemical impedance spectroscopy is used to test the reversible growth of the polymeric film. By analyzing the three-dimensional Nyquist plots at different potentials during the discharge/charge process, detailed electrochemical reaction information can be obtained, which can further verify the reversible formation of a polymeric film at low potential.

## Introduction

With the rapid development of electric vehicles and hybrid electric vehicles, the trend of lithium ion batteries (LIBs) toward achieving higher energy density and greater output power is determined by the electrode materials (Wang et al., 2011; Zhou et al., 2012; Fu et al., 2013). The quality of materials is mainly judged from several aspects, including cycle performance, rate performance, energy density, etc.

For superior cycle performance, theoretically, the cycle curve should remain stable. In practice, this is difficult to achieve. For most electrode materials in LIBs, the capacities decay after repeated cycles. However, in recent years, there are some reports regarding LIB electrode materials which show the opposite trend, i.e., an increase in capacity after repeated cycles (Li et al., 2013; Ao et al., 2017; Cui et al., 2017; Huang et al., 2017; Yuan et al., 2017; Zhang et al., 2018; Zheng et al., 2018). These materials are primarily transition metal oxides and sulfides, and discussions regarding capacity increase focus on the activation of the material and the reversible formation of a gel-film caused by electrolyte decomposition (AbdelHamid et al., 2017; Deng et al., 2017; Li Z. et al., 2017; Tang et al., 2017; Zhu et al., 2017). For example, Ao et al. synthesized a novel honeycomb-like composite composed of carbon-encapsulated SnO_2_ nanospheres for lithium ion and sodium ion batteries (Ao et al., 2017). This composite shows a capacity increase at a rate of 500 mA g^−1^ in LIBs, and the enhancement was assigned to the activation process of the SnO_2_-based electrodes. Yuan et al. fabricated SnO_2_/polypyrrole hollow spheres by a liquid-phase deposition method using colloidal carbon spheres as a template, and the composite electrode showed a significant increase from 404 to 899 mAh g^−1^ (Yuan et al., 2017). This phenomenon was explained by the continuously reversible formation of a polymeric gel-like film. Similarly, Abdel Hamid et al. prepared iron oxide/rGO and SnO_2_/rGO nanosheets for use in LIBs, and these electrodes also show capacity increase (AbdelHamid et al., 2017). From these examples, we can see that reversible polymeric gel-like films greatly increase the electrode capacity, and sometimes the increased capacity exceeds the theoretical capacity of the material.

In this paper, Cu_2_Se nanoflake arrays supported by Cu foams were prepared by a simple hydrothermal method. Cu_2_Se is an important and meaningful material in the field of energy storage and conversion, such as solar cell, thermoelectric, oxygen reduction reaction, sodium, and lithium ion battery et al. (Xue et al., 2006; Liu et al., 2013; Nguyen et al., 2013; Li et al., 2014; Ge et al., 2018c) For LIBs, the Cu_2_Se electrode shows interesting cycle performance: the capacity after the 20th cycle is 213.4 mAh g^−1^, and the capacity of the 200th cycle is 736.7 mAh g^−1^, which is about 3.5 times the previous capacity. The capacity increase was discussed and electrochemical impedance spectroscopy (EIS) was used to understand the physical mechanisms governing the observed capacity increase.

## Experiment

### Synthesis of Cu_2_Se electrode

Typical synthesis of Cu_2_Se supported by Cu foam is as follows. CH_4_N_2_Se (Selenourea, 30 mg) and 40 mL DI water were mixed under stirring. After stirring, it was transferred into a Teflon-lined stainless steel autoclave with 50 mL capacity. A piece of cleaned Cu foam (110 PPI pore size and 1.6 mm thick) with an area of 4.0 × 2.0 cm^2^ was put into the autoclave as both a reactant and substrate. The autoclave was maintained in an oven at 140°C for 12 h. After the autoclave was allowed to cool to room temperature, the Cu foam piece with the synthesized Cu_2_Se was fetched out and rinsed with deionized water and ethanol several times, and then dried at 60°C in vacuum.

On average, Cu_2_Se mass loading is ~2 mg cm^−2^. By carefully weighing the mass of Cu foam before and after reaction, the active mass of Cu_2_Se (m_CuSe_) was derived from m_CuSe_ = Δm × 240.2/64.13, where Δm is the mass difference of the Cu foam before and after hydrothermal synthesis.

### Structural characterization

The structure and morphology of the Cu_2_Se materials were characterized by X-ray powder diffraction (XRD, Rigaku D/Max-2400 with Cu Kα radiation) and field-emission scanning electron microscopy (SEM, Hitachi, S-4800).

### Electrochemical characterization

Electrochemical characterization was carried out using a CR-2032-type coin cell, which was assembled in a high-purity glove box filled with argon (H_2_O < 0.1 ppm, O_2_ < 0.1 ppm, Mikrouna Co., Ltd.). Cu_2_Se was used as the working electrode and Li foil was used as the counter and reference electrode. Celgard 2320 was used as the separator membrane. The electrolyte was 1 M lithium hexafluorophosphate (LiPF_6_) dissolved in ethylene carbonate: dimethyl carbonate: ethyl methyl carbonate in a 1:1:1 volume ratio. Galvanostatic discharge/charge cycling, cyclic voltammetry, and electrochemical impedance spectroscopy (EIS) were carried out at room temperature using a multichannel battery tester (Neware, BTS-610) and an electrochemical workstation (CHI, 660E), respectively. For EIS test, the frequency range is from 1 to 100,000 Hz, including the middle- and high-frequencies. The test voltage is from 0.02 to 3 V, and the step is 0.1 V. Thirty sets of tests from 0.02 to 3 V are without interruption.

## Results and discussion

**Figure 1:**
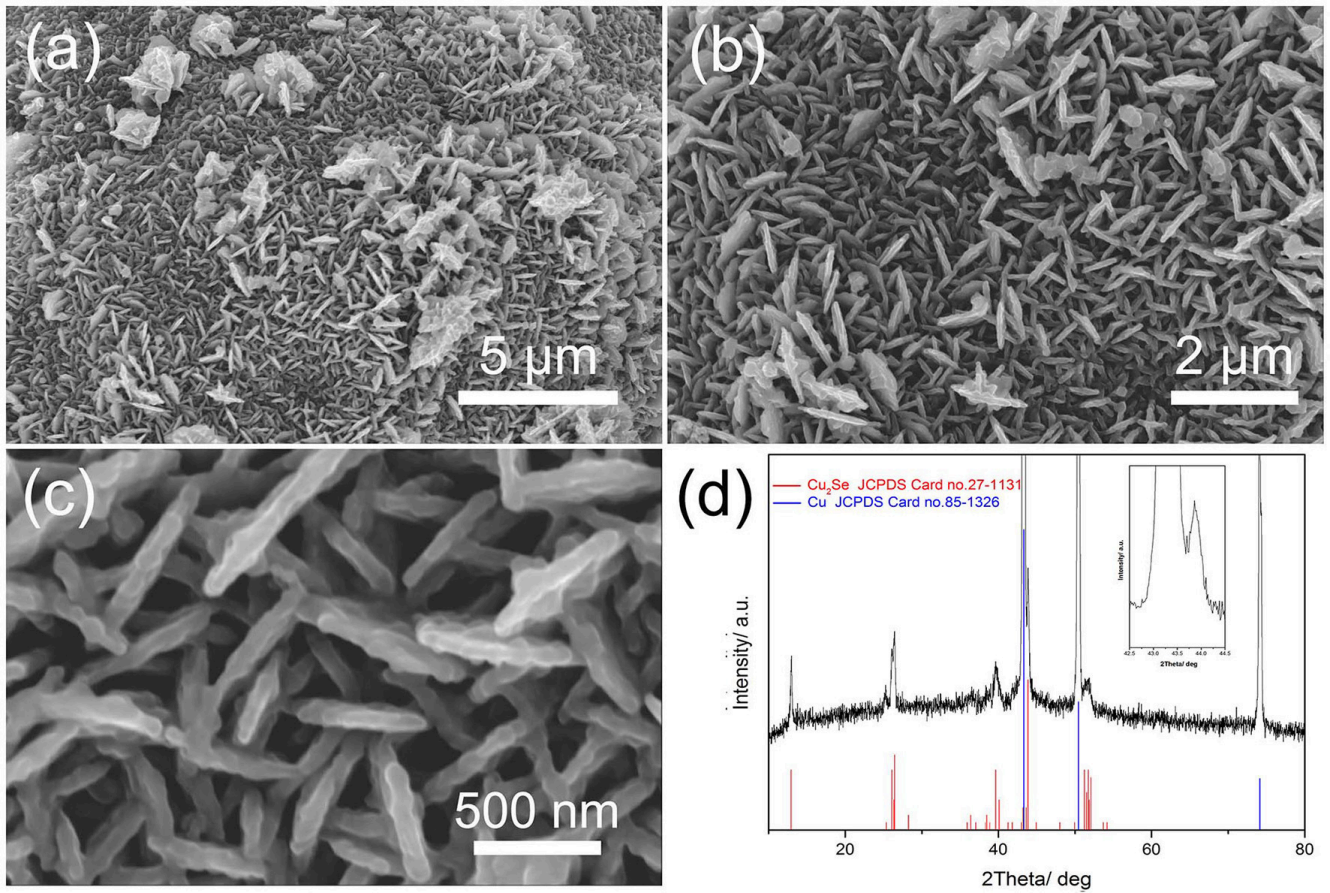
**(a–c)** different-magnification SEM images of Cu_2_Se nanoflakes array supported by Cu nanofoams; **(d)** XRD patterns.

Scanning electron microscope (SEM) images of Cu_2_Se supported by Cu foams after hydrothermal synthesis are shown in Figures 1a–c. A porous nanoflake array structure is found in the surface of the Cu foams. These nanoflakes, which are approximately 100 nm in thickness, are arranged vertically on the substrate and interconnect with each other, forming a 3D porous structure, as shown in Figure 1c. This structure can benefit the transport of lithium ions and electrons thanks to their porous and 3D interconnected characteristics (Zhang et al., 2015). More importantly, the pores in a porous array structure can increase the contact area between material and electrolyte, as well as relieve volume expansion during the discharge/charge process. The as-prepared electrode is characterized by XRD, as shown in Figure 1d. There are three typical diffraction peaks located at 43.3, 50.4, and 74.1°, corresponding to the (111), (200), and (220) faces in Cu, respectively (JCPDS No. 85-1326). Five secondary diffraction peaks can be observed at 13.0, 26.2, 26.5, 39.8, and 44.0°, which match well with (030), (060), (221), (090), and (012) planes of Cu_2_Se, respectively (JCPDS No. 27-1131). These peaks indicate that the material formed during hydrothermal synthesis is Cu_2_Se.

Electrochemical tests were conducted on coin-type cells to evaluate the electrochemical performance of the Cu_2_Se as anodes in LIBs. Figure S1A shows the initial three discharge/charge voltage profiles at 100 mA g^−1^. In the first cycle, there are three main potential plateaus in the discharge curve, located at 1.7, 1.6, and 1.2 V. Correspondingly, the charge curve shows two main potential plateaus at 1.7 and 2.2 V. For the second and third cycles, the number of main discharge and charge potential plateaus is consistent with first cycle. The phenomenon of complex potential plateaus has been reported in the Cu_2_Se, Cu_2−*x*_Se, Cu_*x*_S, and CuS electrodes (Xue et al., 2006; Ni et al., 2012; Chen et al., 2014; Zhou et al., 2014; Li H. et al., 2017). Correspondingly, the CV curves of the Cu_2_Se electrode are tested at a scan rate of 0.1 mV s^−1^ and are shown in Figure S1B. Compared to the three main potential plateaus in the first discharge process, there are only two peaks in the first cathodic scan, which can be attributed to the closeness of the two potential plateaus. Meanwhile, two peaks appear at 1.94 V and 2.23 V during the anodic scan. For the 2nd and 3rd cycles, three reduction peaks near 2.1, 1.7, and 1.5 V and two oxidation peaks at about 1.8 and 2.2 V are observed, which is consistent with the results from the 2nd and 3rd discharge/charge voltage profiles.

**Figure 2:**
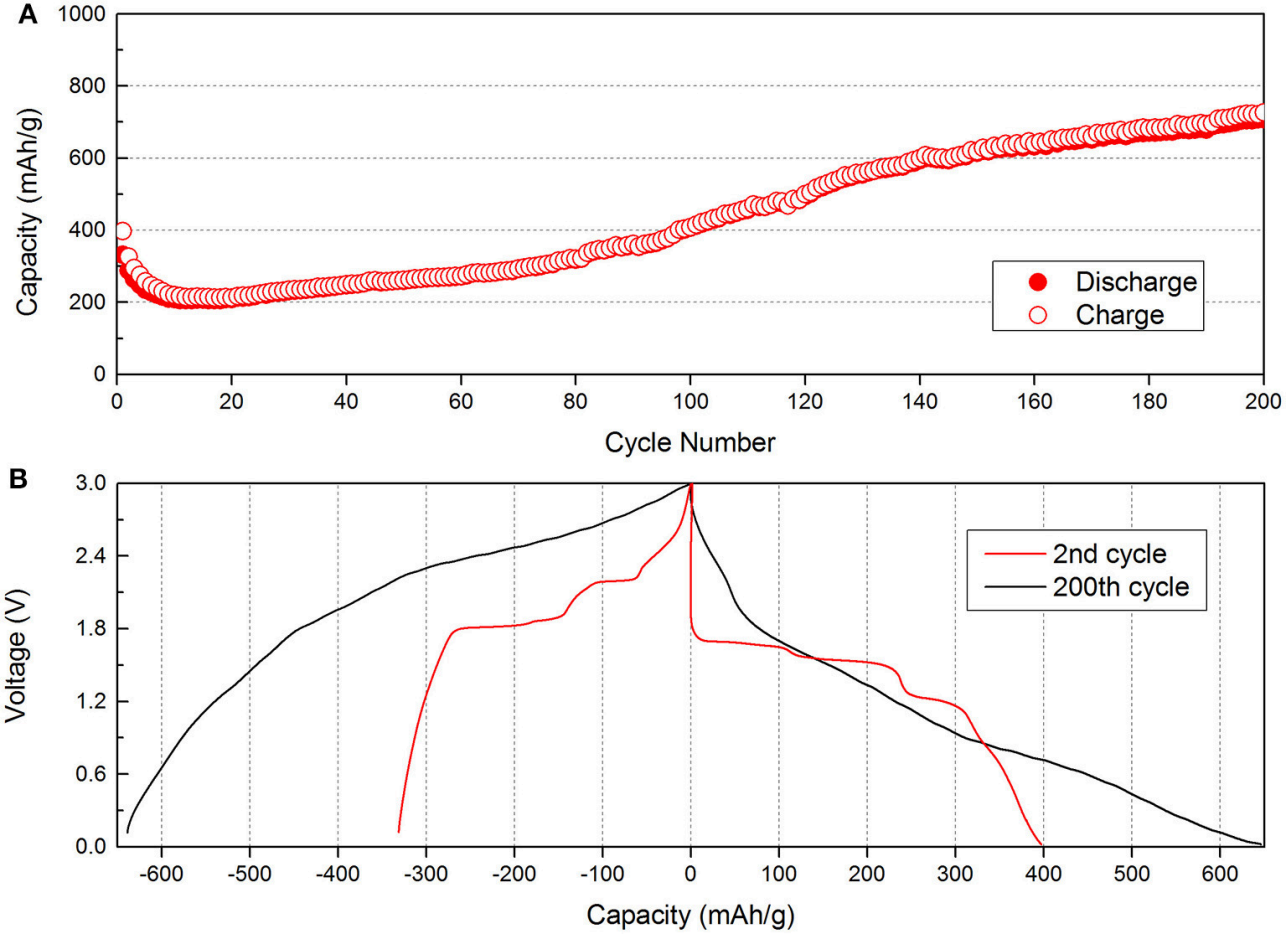
Electrochemical performances of the Cu_2_Se electrode: **(A)** Cycling performance at a rate of 100 mA g^−1^; **(B)** the 2nd and 200th discharge/charge voltage profiles.

Figure 2A shows the cycling performance of Cu_2_Se nanoflake electrode at a current density of 100 mA g^−1^. The first and second discharge capacities are 397.3 and 326.8 mAh g^−1^, respectively. During the first 20 cycles, the capacity decreases to 213.4 mAh g^−1^. However, after the 20th cycle, the capacity increases quickly. Compared to the 20th discharge capacity, the capacity of 200th cycle is 736.7 mAh g^−1^, which is about 3.5 times the previous capacity. This capacity far exceeds its theoretical capacity (260 mAh g^−1^). The phenomenon of increased capacity has been found in many metal oxide, sulfide, and selenide materials, and a lot of useful discussions have been reported (Ge et al., 2018a). There are two general explanations: One is the activation of materials during cycling; the other is the formation of a gel film caused by decomposition of the electrolyte. However, for this electrode, the main reason for the capacity increase cannot be the activation of the material as the capacity increase from 213.4 to 736.7 mAh g^−1^ (i.e., from 0.795 to 2.385 mAh) is much higher than its theoretical value. In order to determine the real reason for the capacity increase, contrast voltage profiles curves of the 2nd and 200th discharge/charge are gathered and are shown in Figure 2B. We find that the voltage profiles of the 2nd cycle show plateau features, which corresponds to electrochemical reactions. After the capacity increase, the voltage profiles of the 200th cycle show significant changes and the plateau feature disappears. Figure S2 shows the discharge voltage profiles during the cycles. During the initial stages, we can find the plateaus for Cu_2_Se at 1.5 and 0.75 V. With repeated cycling, the plateaus slowly disappear, and the capacity quickly increases. In order to further study the plateau changes, the CV curves after 200 cycles are tested at a scan rate of 0.5 mV s^−1^ between 0.02 and 3.0 V. The CV results are found to be changed compared to those from 3 initial cycles (as shown in Figure S1B). During the cathodic scan, three obvious peaks can be seen at 2.3, 1.4, and 0.6 V. These two peaks at 2.3 and 1.4 V are related to the peaks at 2.1 and 1.7 V of the initial CV curves. However, the largest peak at 0.6 V is a new peak, which cannot be found in the initial CV curves. We can also find a new peak at 2.6 V during the anodic scan. The appearance of two new peaks in the CV curves is caused by the reversible formation of a polymeric gel-film, which has reported to occur in other materials (Han et al., 2014; AbdelHamid et al., 2017; Deng et al., 2017; Huang et al., 2017).

**Figure 3:**
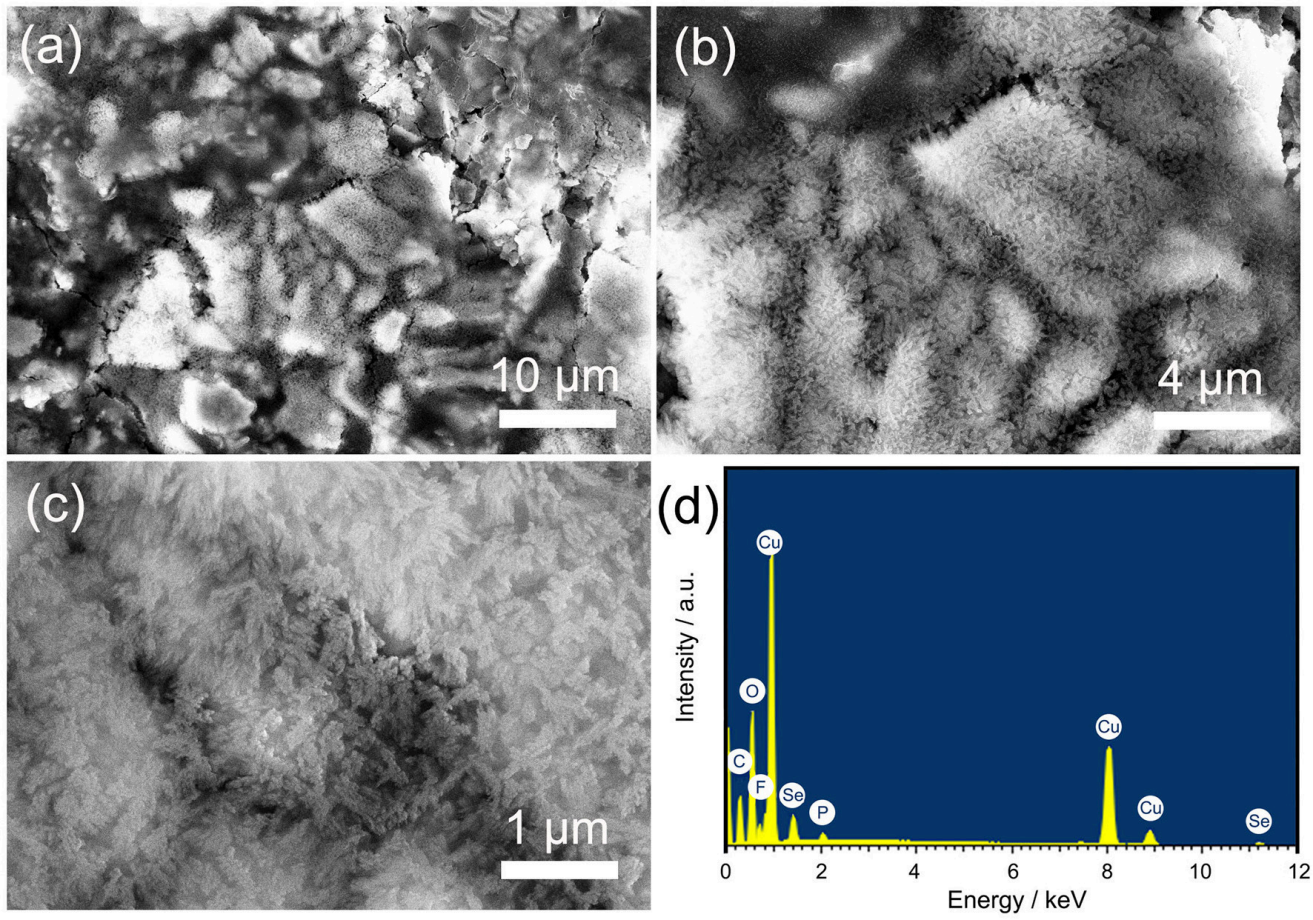
**(a–c)** different-magnification SEM images of Cu_2_Se nanoflakes array after 200 cycles at 0.5C; **(d)** EDS patterns.

As early as 2002, the Tarascon group has systematically studied the problem of capacity increase in metal oxides (Laruelle et al., 2002; Grugeon et al., 2003). Tarascon found that the reversible formation/dissolution of a conducting-type polymeric film at low potential in an alkyl carbonate solution led to the increased capacity and opined that highly reactive pristine metallic nanograins promote the growth of the polymeric film. To prove this point, Cu_2_Se was imaged using SEM after 200 cycles, as shown in Figure 3. It can be seen that a polymeric film layer has grown in most areas of the material surface. According to the SEM characteristics, these polymeric films possess a certain degree of electrical conductivity. In some exposed areas, we can find a large number of nanoparticles with about 10 nm particle size (Figure 3b). This nanoparticle morphology is different from that of the previous nanoflake array, which confirms Tarascon's view. More importantly, compared to the nanoflake structure, a nanoparticle structure has a larger specific surface area. This can provide more locations for growing a reversible polymeric film (RPF), resulting in a greater capacity increase. EDS results show the elemental composition of the sample after 200 cycles. In addition to the copper and selenium elements that came from the original active material, we also found carbon, oxygen, fluorine, and phosphorus elements on the surface of the material. These elements come from the electrolyte and form reversible polymeric and solid electrolyte interface (SEI) films that remain on the surface of the material.

**Figure 4:**
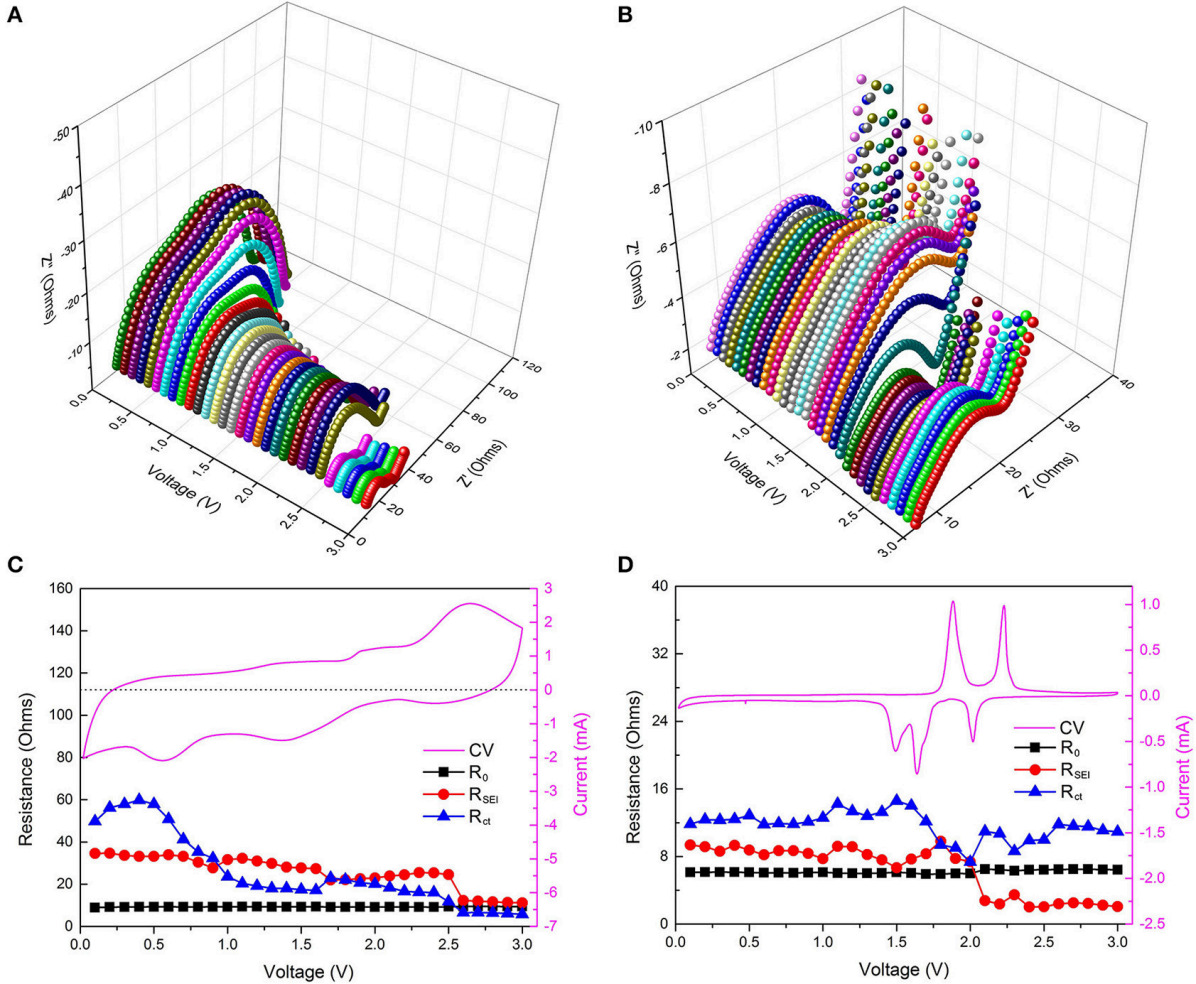
**(A,B)** 3D Nyquist plots at different potentials after 200 cycles and 3 CV cycles; **(C,D)** show the corresponding fitting resistances at different potentials with the CV curves after 200 cycles and 3 CV cycles.

To further verify the reversible growth of RPF, we performed electrochemical impedance spectroscopy (EIS) after 200 cycles and after 3 CV cycles in the Cu_2_Se samples. EIS is an effective electrochemical test technique, which can be used to perform non-destructive testing of the reaction process inside the electrode in real-time (Fattah-alhosseini and Imantalab, 2015; Ge et al., 2018b; Joshi et al., 2018; Peng et al., 2018). We examine the growth of RPF by testing medium- and high-frequency EIS at different potentials (0.02–3 V vs. Li/Li^+^). Figure 4A shows the 3D Nyquist plot at different potentials after 200 cycles. It can be clearly seen that the Nyquist curves are different and continuous at different potentials (0.02–3 V vs. Li/Li^+^), illustrating the difference and continuity of the electrode reaction at different potentials. In the low potential area, the second semicircle significantly increased, proving the growth of the RPF of the electrode material in the low potential area. To the opposite end, the two semicircles at a high potential area of about 2.5–3.0 V are all small, and this phenomenon may be caused by the decomposition of the RPF. This conclusion is consistent with the CV results after 200 cycles (as shown in Figure S2B). Correspondingly, we also tested the EIS after 3 CV cycles at different potentials, and the 3D Nyquist plot is shown in Figure 4B. The Nyquist curves after 3 CV cycles at different potentials are obviously smaller than the curves after 200 cycles, proving that the internal resistance after 3 CV cycles is smaller than the resistance after 200 cycles. This may occur due to decomposition of the electrolyte caused by the formation of RPF after 200 cycles. As can be seen from Figure 4B, the position of the big semicircle is about 1.5–2.0 V, corresponding to the electrode reaction potential. This is because a strong electrochemical reaction occurs at the electrode reaction potential and a large amount of charge is transferred from the ions to the electrons, which results in a large charge transfer resistance to form a larger semicircle (Jeon et al., 2014; Rodrigues et al., 2016; Yang et al., 2017).

To further analyze the EIS results, the obtained impedance data were analyzed by fitting the equivalent electrical circuit (as shown in Figure S3). R_0_, R_SEI_, and R_ct_ are the electrolyte resistance, SEI film resistance, and charge transfer resistance, respectively. The fitted impedance parameters after 200 cycles and after 3 CV cycles are listed in Figures 4C,D, respectively. For ease of understanding, the respective CV curves were added in the right-Y scale, and detailed numerical comparison of the impedance is listed in Table S1. From Figure 4D, we can see that the resistances of R_0_, R_SEI_, and R_ct_ in the sample after 3 CV cycles at different potentials are relatively stable (these resistance values are < 15 ohms), indicating that the active material/SEI film/electrolyte film has a stable structure under different charging and discharging potentials. More importantly, the stable material structure proves that there is no formation of RPF during the discharge/charge process, which is consistent with the CV curve in Figure 4D. Compared to the sample tested after 3 CV cycles, the resistances after 200 cycles at different potentials are more complicated. First, the electrolyte resistances after 200 cycles are about 9.2 ohms, which is higher than the sample after 3 CV cycles (6.0 ohms) and is caused by electrolyte reduction due to the decomposition of the electrolyte after 200 cycles. Second, the SEI film resistances after 200 cycles at different potentials are between 10 and 40 ohms, which are also much higher than that of the sample after 3 CV cycles (as shown in Table S1). The high SEI film resistance may be caused by the irreversible accumulation of a polymeric film after 200 discharge/charge cycles, as can be seen from Figure 3. Finally, the charge transfer resistances after 200 cycles vary greatly with the potential. The highest resistance can reach up to 59.81 ohms at 0.4 V, and the lowest resistance is about 5.74 ohms at 3.0 V. Charge transfer resistance is related to the electrochemical reaction, and in the discharge/charge process of Cu_2_Se after 200 cycles, there are two electrochemical reactions. One is the redox reaction of Cu_2_Se, and the other is the synthesis and decomposition reaction of RPF (Yuan et al., 2009). According to relevant reports and previous analysis, the synthesis reaction of RPF for Cu_2_Se electrode occurs in the low potential area (Grugeon et al., 2003; Ponrouch et al., 2012; Li et al., 2013). In this potential area, the RPF synthesis provides additional capacity. Therefore, the charge transfer reaction occurs in the RPF between the electrode and the SEI film instead of within the electrode. With the continuous formation of the RPF, the thicknesses of the SEI film and the RPF increase, and the charge transfer resistance sharply increases. At the same time, the SEI film resistance also increases accordingly. In the high potential area, the corresponding electrochemical reaction is the decomposition of the RPF. As the film decomposes, the charge transfer resistance decreases, and the SEI film resistance also decreases, which is consistent with the CV curve in Figure 4C. As shown in Table S1, one can see that, whether we consider SEI film resistance or charge transfer resistance, they have the smallest difference in the high potential area. These EIS results show that the RPF grows in the low potential region and decomposes in the high potential region.

## Conclusion

In this paper, we prepared Cu_2_Se nanoflake arrays through a facile hydrothermal method, and the synthesized Cu_2_Se material was used as an anode for lithium ion batteries, which shows superior cycle performance with high capacity. The capacity increase was discussed and electrochemical impedance spectroscopy was used to test the reversible growth of a polymeric film. By analyzing the Nyquist plots at different potentials during discharge/charge cycles, we can obtain detailed electrochemical reaction information and further verify that the reversible formation of a polymeric film at low potential leads to the observed capacity increase.

## Author contributions

XL is responsible for experimental design, ZL is responsible for data analysis, ZZ and CL are responsible for instrument operation, and XL and ZL are responsible for writing article.

### Conflict of interest statement

The authors declare that the research was conducted in the absence of any commercial or financial relationships that could be construed as a potential conflict of interest.
