# Supplementary material for: Capacity Increase Investigation of Cu2Se Electrode by Using Electrochemical Impedance Spectroscopy
Source: Front Chem. 2018 Jun 12;6:221. doi: 10.3389/fchem.2018.00221 (PMC6005858; doi:10.3389/fchem.2018.00221)
Supplement: Supplementary file 1 [file Presentation_1.PDF]

Supporting Information

Investigation of capacity increase of Cu2Se electrode in lithium ion batteries using electrochemical impedance spectroscopy

Xiuwan Li*, Zhixin Zhang, Chaoqun Liu, Zhiyang Lin

Fujian Provincial Key Laboratory of Light Propagation and Transformation, College of Information Science and Engineering, Huaqiao University, Xiamen 361000, China

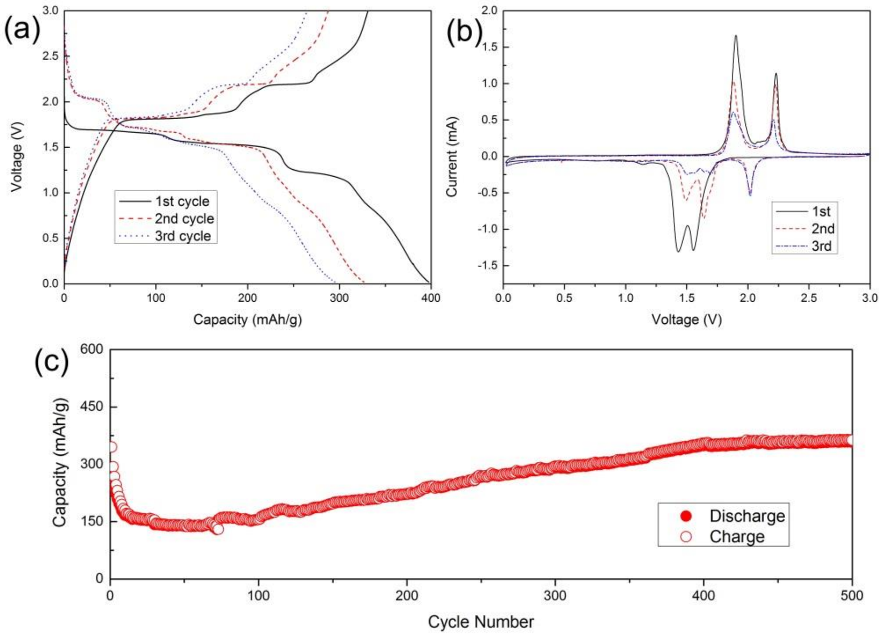

Fig.S1 Electrochemical performances of the Cu_2Se electrode: (a) Discharge/charge voltage profiles and (b) Cyclic voltammograms for the initial three cycles; (c) Cycling performance at a rate of 200 mA g^{-1}.

Fig. S1c shows the cycling performance of Cu_2Se nanoflake electrode at a current density of 200 mA g^{-1}. The first and second discharge capacities are 345.3 and 290.5 mAh g^{-1}, respectively. During the first 50 cycles, the capacity decreases to 137.5 mAh g^{-1}. However, the capacity increases quickly after this cycle. Compared to the 50th discharge capacity, the capacity of 500th cycle is 363.1 mAh g^{-1}, which show the same trend of capacity increase.

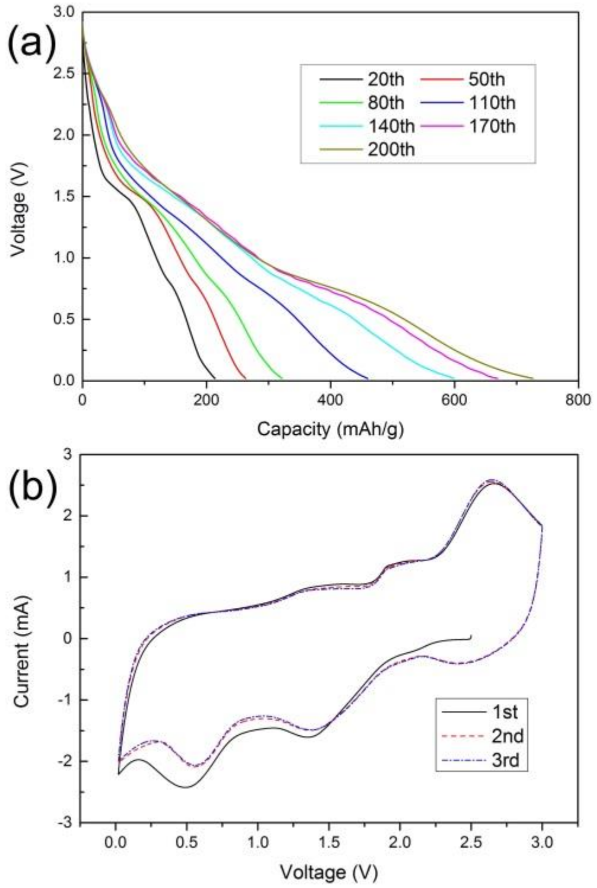

Fig.S2 (a) Discharge voltage profiles at different cycles; (b) Cyclic voltammograms after 200 cycles at 100 mA g^{-1}.

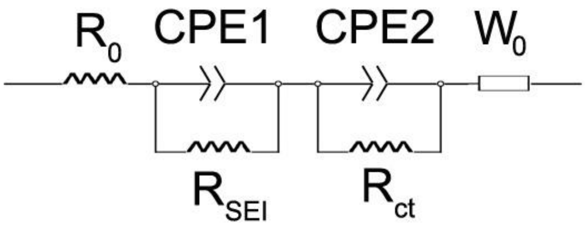

Fig.S3 The equivalent electrical circuit for electrochemical impedance spectroscopy.

Table.S1 The detailed numerical comparison of the fitting impedance at different potentials for electrolyte resistance, SEI film resistance, and charge transfer resistance.

**Potential**	**electrolyte resistance (Ω)**		**SEI film resistance (Ω)**		**charge transfer resistance (Ω)**	
**(V *vs.* Li/Li$^+$)**	3 CV	200 cycles	3 CV	200 cycles	3 CV	200 cycles
**0.1**	6.18	8.928	9.361	34.54	11.83	49.74
**0.2**	6.139	9.168	9.154	34.64	12.37	56.22
**0.3**	6.155	9.173	8.629	33.62	12.31	57.92
**0.4**	6.145	9.258	9.323	33.15	12.41	59.81
**0.5**	6.123	9.287	8.755	33.15	12.86	57.91
**0.6**	6.070	9.270	8.205	33.92	11.80	50.80
**0.7**	6.071	9.226	8.690	33.21	11.93	41.22
**0.8**	0.062	9.251	8.681	30.37	11.83	35.24
**0.9**	6.085	9.303	8.360	27.75	12.11	32.26
**1.0**	6.134	9.286	7.732	31.57	12.58	23.59
**1.1**	6.032	9.334	9.213	32.29	14.22	20.34
**1.2**	5.998	9.353	9.164	30.93	13.35	19.20
**1.3**	6.003	9.298	8.222	29.80	12.79	18.04
**1.4**	6.043	9.273	7.588	28.10	13.26	18.01
**1.5**	6.129	9.286	6.669	27.71	14.58	17.42
**1.6**	6.034	9.317	7.683	27.31	14.02	17.12
**1.7**	5.915	9.207	8.317	22.04	12.15	22.95
**1.8**	5.919	9.218	9.797	22.13	9.386	22.12
**1.9**	5.995	9.233	7.765	22.64	9.023	20.70
**2.0**	5.974	9.212	7.402	22.97	7.347	20.10
**2.1**	6.494	9.237	2.775	23.92	10.96	18.34
**2.2**	6.459	9.262	2.383	24.48	10.70	16.65
**2.3**	6.304	9.178	3.471	25.52	8.625	16.22
**2.4**	6.417	9.128	2.023	25.41	9.899	16.06
**2.5**	6.428	9.346	2.047	24.55	9.989	11.77
**2.6**	6.456	9.487	2.402	12.25	11.80	6.554
**2.7**	6.486	9.540	2.512	12.04	11.61	6.586
**2.8**	6.495	9.503	2.435	11.68	11.54	6.398
**2.9**	6.460	9.448	2.238	11.38	11.09	6.132
**3.0**	6.427	9.405	2.064	11.20	10.93	5.745
